# Neogenin expression in ependymo-radial glia of the larval sea lamprey *Petromyzon marinus* spinal cord.

**DOI:** 10.17912/micropub.biology.000810

**Published:** 2023-04-17

**Authors:** Laura González-Llera, Michael I. Shifman, Antón Barreiro-Iglesias

**Affiliations:** 1 Department of Functional Biology, CIBUS, Faculty of Biology, Universidade de Santiago de Compostela, Santiago de Compostela, Galicia, Spain; 2 Neuroscience Consulting, LLC.

## Abstract

Neogenin is a receptor mainly known for its roles during axon pathfinding. However, neogenin is expressed in neuronal precursors of ventricular and subventricular zones of the nervous system and recent work has shown that it regulates adult neurogenesis. Here, we generated an antibody against the sea lamprey neogenin to study its expression in the larval spinal cord. Immunofluorescence experiments show that neogenin is expressed in ependymo-radial glial cells (ERGs) located in the ependymal region of the central canal of mature larval sea lampreys. Our results provide a basis for the future study of the role of neogenin in lamprey ERGs.

**Figure 1. Neogenin (NEO) expression in spinal cord ERGs of mature larval sea lampreys.**
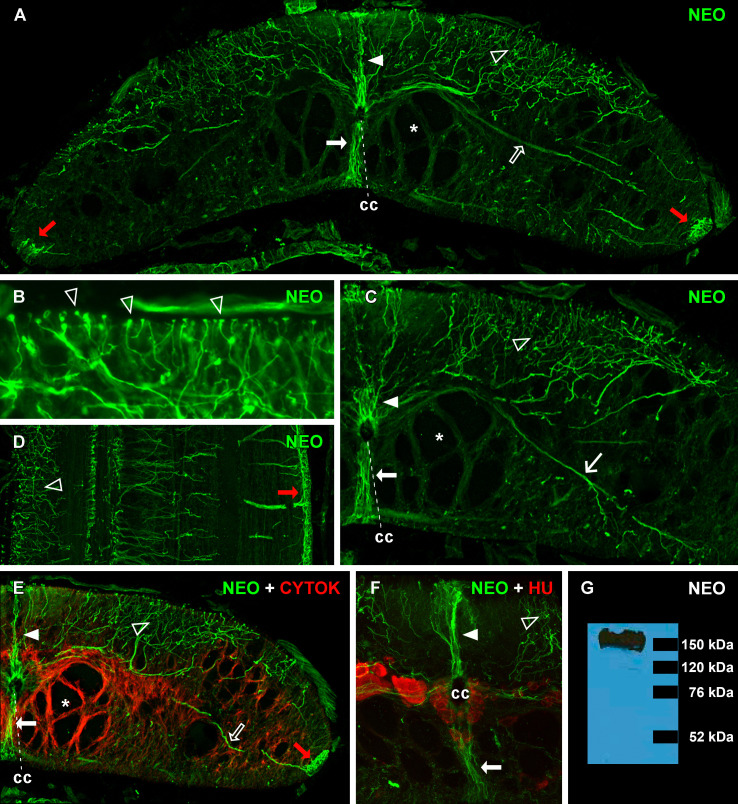
( **A** ) Transverse section of the larval spinal cord (at the level of the 5 ^th^ gill opening) showing the presence of neogenin-ir ERGs. The cell bodies of neogenin-ir ERGs are located in the ependymal layer of the central canal (cc) and their radial processes end at the pial surface. ( **B** ) Detail of the dorsal region of the spinal cord showing the presence of endfeet-like structures of the radial processes of dorsolateral ERGs. ( **C** ) Detail of a transverse section of the spinal cord showing the thick radial projection of ventrolateral ERGs ending at the ventral surface of the cord (thin arrow). Note that this radial projection leaves the ependymal region over the conspicuous bundle of giant descending axons (asterisks). ( **D** ) Horizontal section of the spinal cord showing how the endings of radial processes from lateral ERGs cover the entire edge of the spinal cord (red arrows). ( **E** ) Double immunofluorescence showing the lack of cytokeratin (CYTOK) expression (red) in larval sea lamprey ERGs. Note that cytokeratin immunoreactivity is only observed in free astrocytes of the spinal cord parenchyma and how astrocytic processes surround the giant descending axons. ( **F** ) Double immunofluorescence showing the lack of HuC/HuD (HU) expression (red) in neogenin-ir ERGs. HuC/HuD immunoreactivity is only observed in spinal cord neurons. ( **G** ) Western Blot showing that the anti-neogenin antibody only recognizes a single band of approximately 150 KDa (see the corresponding black bar) in central nervous system (CNS) protein extracts of the sea lamprey. Arrowheads indicate the radial processes of dorsal ERGs. Empty arrowheads indicate the radial processes of dorsolateral ERGs. Empty arrows indicate the thick projection of lateral ERGs ending at the spinal cord edge. Thick arrows indicate the radial processes of ventral ERGs.

## Description

In the 90s, Hedgecock and co-workers showed that mutations in *Caenorhabditis elegans* genes unc-5, unc-6 and unc-40 caused uncoordinated phenotypes because of disruptions in axon pathfinding and cell migration (see De Vries and Cooper, 2008). unc-6 has been found to encode a secreted protein (netrin) that acts either as an attractive or repulsive guidance cue. Netrin/UNC-6 chemoattraction is mediated by UNC-40, while UNC-5 alone or a receptor complex of UNC-5/UNC-40 mediates chemorepulsion. Now, the orthologues of these molecules have been identified in *Drosophila melanogaster* , zebrafish, *Xenopus laevis* , chicken, mammals (see Moore et al., 2007) or in lampreys (Shifman and Selzer, 2000; Shifman et al., 2009; Barreiro-Iglesias et al., 2012) . While there is one member of the UNC-40 receptor family in invertebrates, vertebrates have two orthologs: deleted in colorectal cancer (DCC) and neogenin; both of which act as receptors for netrin (see De Vries and Cooper, 2008). The repulsive guidance molecule (RGM) family of proteins (Rajagopalan et al., 2004) , bone morphogenetic proteins (BMPs; Hagihara et al., 2011) or draxin (Ahmed and Shinmyo, 2021) are also ligands for neogenin.

Neogenin regulates many cellular processes in the nervous system, where it mediates both attractive and repulsive axonal guidance. Neogenin mediates RGM induced repulsion of temporal retinal axons navigating through the chick tectum (Monnier et al., 2002) . In *Xenopus* embryos, axons expressing neogenin and navigating along the supraoptic tract are attracted by a source of netrin-1 and repulsed by RGMa (Wilson and Key, 2006) . Neogenin and RGMa interaction is required to establish neuroepithelial cell polarity during neural tube formation (Mawdsley et al., 2004; Kee et al., 2008) . Neogenin acting as dependence receptor also regulates neuronal survival (Fujita et al., 2008; Shabanzadeh et al., 2015) . Interestingly, neogenin is also found on dividing neurogenic and gliogenic precursors of ventricular zones throughout the embryonic CNS and is also observed on adult neural stem cell populations residing in the forebrain subventricular zone (Vielmetter et al. 1994; Keeling et al. 1997; Fitzgerald et al. 2006, 2007; Bradford et al., 2010) . In mammals, neogenin expression is weak in the earliest developing CNS but its expression is broadened and intensified as neurogenesis proceeds (Gad et al., 1997) . In zebrafish embryos, neo1a (one of the zebrafish homologues of neogenin) mRNA expression has been also detected in proliferative ventricular zones (Shen et al., 2002) . Recent work has also shown that neogenin regulates adult neurogenesis (O`Leary et al., 2015; Sun et al., 2018; Isaksen and Yamashita, 2020).

Lampreys are a model of interest in comparative studies of the nervous system because of their key phylogenetic position as jawless vertebrates and because of their amazing capacity for spontaneous regeneration after spinal cord injury (see Rodicio and Barreiro-Iglesias, 2012; Sobrido-Cameán and Barreiro-Iglesias, 2022; González-Llera et al., 2022). The neogenin transcript has been previously identified in the sea lamprey (Shifman et al., 2009) , and its expression has been studied by means of in situ hybridization in giant descending neurons of the sea lamprey brainstem (Shifman et al., 2009; Chen et al., 2017; Chen and Shifman, 2019) . After a complete spinal cord injury in lampreys, neogenin appears to be preferentially expressed in giant descending neurons that are considered “bad regenerators” (i.e., neurons with low intrinsic capacity for axonal regeneration) (Shifman et al., 2009) . Indeed, neogenin knockdown with morpholinos promotes the survival and regeneration of giant descending neurons after a complete spinal cord injury in larval lampreys (Chen et al., 2017; Chen and Shifman, 2019) . However, the expression of neogenin has not been yet studied in the sea lamprey spinal cord. Here, we aimed to generate an antibody against sea lamprey neogenin and study its expression in the spinal cord of mature larval sea lampreys by using immunofluorescence methods and confocal microscopy.

As can be observed in transverse sections of the spinal cord, the anti-neogenin antibody only labelled the ERG cells surrounding the central canal of the spinal cord ( Fig. 1A -F). These glial cells have been also called ependymal cells (Nieuwenhuys and Nicholson, 1998) or tanycytes (Tretjakoff, 1909) in the sea lamprey spinal cord. The cell bodies of these neogenin-immunoreactive (-ir) ERGs are located in the ependymal layer of the central canal and their radial processes abut in the pial surface of the spinal cord with endfeet-like structures ( Fig. 1B ). We opted for the term ERG because it is being used in modern studies in the spinal cord of mature fish to refer to cells that share characteristics of ependymal and radial glial cells (see Becker and Becker, 2015).

Based on the projections of the radial processes of neogenin-ir ERGs, we can distinguish the presence of 5 subpopulations of ERGs: a dorsal population with processes directed to the dorsal midline ( Fig. 1A, C, E and F ), dorsolateral cells with radial processes directed to the dorsal column and the dorsolateral region of the spinal cord ( Fig. 1A -F), lateral cells that form a conspicuous radial projection ending at the lateral edge of the spinal cord ( Fig. 1A, D and E ), a ventrolateral population with radial processes ending in the ventrolateral region of the spinal cord ( Fig. 1C ) and, finally, a ventral group of cells with radial processes directed towards the ventral midline ( Fig. 1A, C, E and F ). The conspicuous projection of lateral ERGs leaves the ependymal region over the main group of giant descending axons ( Fig. 1A ). Horizontal sections reveal how the endings of this prominent projection from lateral neogenin-ir ERGs cover the lateral edges of the spinal cord ( Fig. 1D ).

Double-labelling immunofluorescence experiments with the LCM29 antibody showed that the neogenin-ir ERGs do not express cytokeratins, which are only expressed in free astrocytes of the spinal cord parenchyma and in the astrocytic processes surrounding giant descending axons ( Fig. 1E ). Moreover, additional double-labelling immunofluorescence experiments did not reveal colocalization of HuC/HuD (pan-neuronal marker) immunoreactivity in neogenin-ir ERGs ( Fig. 1F ). Previous studies have shown that lamprey ERGs express GFAP (Wasowicz et al., 1994) or taurine (Shupliakov et al., 1994) . GFAP expression is a common feature with ERGs in jawed vertebrate species (see Becker and Becker, 2015). Interestingly, recent work has shown that taurine levels increase in the lamprey spinal cord after injury and that a taurine treatment promotes axon regeneration in descending neurons of lampreys (Sobrido-Cameán et al., 2020). So, it will be of interest to study the role of neogenin-ir ERGs in axon regeneration in lampreys.

After a complete spinal cord injury in lampreys, there is an increase in cell proliferation, especially in the ependymal region of the central canal (Zhang et al., 2014) . In zebrafish, ERGs activate a program of regenerative neurogenesis after a complete spinal cord injury, proliferate and generate new neurons (Barreiro-Iglesias et al., 2015; Becker and Becker, 2015) . Therefore, our expression data indicates that neogenin might play a role in spinal cord neurogenesis (constitutive or regenerative) in lampreys. Future work should attempt to decipher the role/s of neogenin in sea lamprey ERGs.

## Methods

Animals

Wildtype larval lampreys ( *Petromyzon marinus* L.), 10–14 cm in length (4 to 7 years old) were obtained from streams feeding Lake Michigan and maintained in freshwater tanks at 16°C with the appropriate ventilation conditions until the day of use. Experiments were approved by the Institutional Animal Care and Use Committee at Temple University. Larval sea lampreys (n = 14) were anesthetized by immersion in 0.1% tricaine methanesulfonate (Sciencelab, Houston, TX, USA) before all experimental procedures.

Generation of a sea lamprey specific anti-neogenin antibody

A rabbit polyclonal antibody against sea lamprey neogenin was generated by Pocono Rabbit Farm & Laboratory Inc. (PRF&L; Canadensis, PA, USA) by using as immunogen a peptide synthesized by the same company (peptide sequence: Cys Ser Ala Val Glu Arg Asp Ala Thr His Glu Glu Glu Ile; PRF&L Reference LS2099) coupled to KLH (the Cys residue was added to allow for KLH coupling). The peptide sequence corresponds to a portion of the neogenin protein located in the intracellular region containing the P domains of all 4 neogenin splicing variants found in the sea lamprey (GenBank accession numbers: XM_032947567.1, XM_032947737.1, XM_032947835.1, XM_032947655.1). The antibody was affinity purified from Rabbit 22140 using 25 mL of sera from the final bleed. The affinity column was built using the neogenin peptide synthesized by PRF&L. Final antibody concentration was 0.25 mg/mL, the volume of anti-neogenin antibody was 27 mL and 0.02% Sodium Azide and 1% BSA were added as preservatives.

Immunofluorescence

The body region between the 4th and 7th gill openings was fixed in 4% paraformaldehyde in phosphate buffered saline pH 7.4 (PBS) by immersion for 4 to 5 hours. The tissue was rinsed in PBS, cryoprotected by immersion in 30% sucrose in PBS overnight, and then embedded in Tissue Tek (Sakura, Torrance, CA, USA), frozen and cut in horizontal or transverse planes on a cryostat (18 or 20 µm thick). For neogenin immunofluorescence, the sections were incubated with the rabbit polyclonal antibody against sea lamprey neogenin (see above; dilution 1:200), for 72 hours at 4ºC. A goat anti-rabbit immunoglobulin G (IgG; H +L) antibody (diluted 1:100; Vector, Burlingame, CA, USA; Cat.# DI-1488-1.5) coupled to Dylight 488 was then applied for 1 h at room temperature. Dylight 488 emits green fluorescence (518 nm) after excitation with blue light at a maximum wavelength of 493 nm. All antibody dilutions were carried out in PBS containing 15 % normal goat serum and 0.2 % Triton X-100. Some samples were used for double immunostaining against neogenin and three glial intermediate filament polypeptides of the sea lamprey spinal cord (LCM29 antibody; Merrick et al., 1995) or the pan-neuronal markers HuC/HuD. Sections were incubated overnight at room temperature with a cocktail of the rabbit polyclonal anti-neogenin antibody and the mouse monoclonal LCM29 antibody (dilution 1:100; Merrick et al., 1995) or a mouse monoclonal anti-HuC/HuD antibody (16A11; dilution 1:200; ThermoFisher, Waltham, MA, USA; Cat.# A-21271). Then, the sections were incubated with a cocktail of the goat anti-rabbit antibody (see above) and a donkey anti-mouse IgG (H+L) antibody coupled to Alexa 594 (ThermoFisher; diluted 1:100; Cat#R37115) for 1 hour at room temperature. Alexa 594 emits red fluorescence (617 nm) after excitation with green light at a maximum wavelength of 590 nm. The antibodies were diluted with PBS containing 15% normal goat serum, 15% normal donkey serum and 0.2% Triton X-100. After antibody incubations, the sections were washed in PBS and distilled water, allowed to dry for 30 min at 37 ºC, and mounted in MOWIOL® 4-88 (Merck, Darmstadt, Germany).

Antibody characterization

Specificity of the anti-neogenin antibody was tested by Western blot using larval sea lamprey total protein from CNS homogenates. For extract preparation, the CNS was lysed in TNN buffer (50mM Tris, 150 mM NaCl and 0.5 NP-40 with protease inhibitor cocktail; Sigma). The protein concentration was measured by the Bradford assay, 30 µg lysate was loaded per well for SDS-PAGE and proteins were transferred onto polyvinylidene fluoride (PVDF) or nitrocellulose membranes. Western analysis was conducted by incubation with the anti-neogenin antibody (1:1000; see above) overnight at 4ºC, followed by HRP-tagged secondary anti-rabbit antibody (1:5000; 1 h at room temperature; Americana Qualex, San Clemente, CA, USA). Proteins were visualized by ECL (GE Healthsciences, Piscataway, NJ, USA). The antibody recognized a single band of about 150 kDa ( Fig. 1G ), which is very close to the predicted molecular size for the sea lamprey neogenin amino acid sequences (153 to 155 kDa; ExPASy Proteomics Server; Swiss Institute of Bioinformatics). In addition, no staining was observed in immunofluorescence experiments when the antibody was pre-adsorbed with the immunizing peptide overnight at a concentration of 0.1 mg/mL before incubation with the spinal cord sections.

The generation and specificity of the LCM29 antibody against three major glial intermediate filament polypeptides (molecular weights between 45 and 56 kDa) of the sea lamprey CNS has been previously reported (Merrick et al., 1995) . The 3 sea lamprey glial intermediate filament polypeptides appear to be similar to simple epithelial cytokeratins (Merrick et al., 1995) .

The mouse monoclonal anti-HuC/HuD antibody was raised against the neuronal proteins HuC and HuD, it binds specifically to antigens present exclusively in neuronal cells and is thus useful as marker of neurons in tissue. This antibody has been commonly used in lampreys as a pan-neuronal marker (Fernández-López et al., 2014; Zhang et al., 2014). 

Imaging 

Photomicrographs were taken with a Nikon C2 confocal microscope (20x or 40x objectives). Images were imported into Adobe Photoshop 2022 (Adobe Inc., Mountain View, CA, USA) and minimally adjusted for brightness and contrast, and labels were added.
